# Umbilical cord-derived mesenchymal stem cells cultured in the MCL medium for aplastic anemia therapy

**DOI:** 10.1186/s13287-023-03417-1

**Published:** 2023-08-30

**Authors:** Chuan He, Chao Yang, Qiang Zeng, Zhigang Liu, Fangfang Wang, Qiang Chen, Ting Liu

**Affiliations:** 1 Department of Hematology and Institute of Hematology, West China Hospital, Sichuan University, Chengdu, 610041 China; 2 Stem Cells and Regenerative Medicine Research Center, Sichuan Stem Cell Bank/Sichuan Neo-Life Stem Cell Biotech Inc., 15 Jinquan Road, Chengdu, 610036 China

**Keywords:** Umbilical cord, Mesenchymal stem cell, Wharton's jelly, Aplastic anemia

## Abstract

**Background:**

Mesenchymal stem cells (MSCs) are a class of adult stem cells with self-renewal and multidirectional differentiation potential that may be a treatment for aplastic anemia (AA).

**Method:**

Umbilical cord-derived MSCs were cultured in three media (Mesencult-XF, MCL, and StemPro MSC SFM CTS). HGF, PGE2, ANG-1, TGF-β1, IFN-γ, and TNF-α were detected using ELISA. The AA mouse model was built via post-irradiation lymphocyte infusion. After different treatments, routine blood, VEGF, and Tregs were detected every week. On day 28, all mice were killed, and their femurs were stained with HE.

**Results:**

Umbilical cord-derived MSCs cultured in the three media all conformed to the general characteristics of MSCs. HGF secreted by MSCs in the Mesencult-XF, and MCL was greater than that in the StemPro MSC SFM CTS; ANG-1 and TGF-β1 in the MCL were more than that in Mesencult-XF and StemPro MSC SFM CTS; PGE2 in the MCL and StemPro MSC SFM CTS was more than that in the Mesencult-XF. MSCs in the MCL and StemPro MSC SFM CTS inhibited IFN-γ and TNF-α more than those in the Mesencult-XF. The peripheral blood cell in the AA groups was at a low level while that in the MSC group recovered rapidly. The Treg ratio and VEGF level in the MSC group were higher than those in the AA group. The bone marrow (BM) recovered significantly after MSC infusion.

**Conclusion:**

MSCs in the MCL were advantageous in supporting hematopoiesis and modulating immunity and had the potential for effective treatment of AA.

**Supplementary Information:**

The online version contains supplementary material available at 10.1186/s13287-023-03417-1

## Introduction

Mesenchymal stem cells (MSCs) are a population of adult stem cells with the potential for self-renewal and multidirectional differentiation [1]. In recent studies, MSCs were found to have potent immunomodulatory activities, which also have potential applications in graft versus host disease (GVHD) and autoimmune diseases [2–5]. MSCs also support hematopoietic stem cells (HSCs) by secreting hematopoietic growth factors and differentiating into osteoblasts, which are important components of the hematopoietic microenvironment, to promote the reconstruction of the hematopoietic microenvironment. Additionally, due to the lack of expression of MHC-II, CD80, CD86, CD40, and CD40L, MSCs are immune tolerant and escape recognition by allogeneic effector T cells [6]. In addition to bone marrow (BM), MSCs are present in many other tissues such as synovium, fat, umbilical cord, cord blood, amniotic membrane, amniotic fluid, and placenta [7, 8]. Among these tissues, umbilical cord-derived cells are easy to collect in large quantities and without ethical issues. MSCs can be obtained from different parts of the umbilical cord [9–13], and Wharton's jelly-derived MSCs have the following advantages: minimal contamination of non-MSCs, maximum quantity through the shortest transmission culture, excellent stemness, and high differentiation potential that are suitable for clinics [14]. In addition to the production site, the proliferation, differentiation, and immune regulation of MSCs are influenced by different serum-free culture systems [15].

Aplastic anemia (AA) is a BM failure syndrome characterized by hypoplasia of the BM and a decrease in whole blood cells in the peripheral blood, which are caused by an increase in interferon-γ (IFN-γ) and tumor necrosis factor-α (TNF-α), and a decrease in regulatory T cells (Tregs). Allogeneic hematopoietic stem cell transplantation (allo-HSCT) and immunosuppression therapy (IST) are the current standards of care for AA. However, 30% of patients remain ineligible for allo-HSCT or experience ineffective treatment or relapsed. It was demonstrated that the hematopoietic microenvironment also played a vital role in maintaining normal hematopoiesis of HSCs. Studies found decreased proliferation and increased apoptosis of MSCs, reduction in MSC differentiation into osteoblasts, and a decrease in stem cell factor (SCF), vascular endothelial cell growth factor (VEGF), transforming growth factor-beta1(TGF-β), and angiogenesis factor-1 (ANG-1) derived from MSCs in AA patients [16–18]. Therefore, MSCs are very important in the formation of a normal hematopoietic microenvironment and in maintaining the "stemness" of HSCs.

Since abnormal MSCs are involved in the complex pathophysiological process of BM hematopoietic failure, transfusion of normal MSCs may replace defective MSCs and complete their immunomodulation and hematopoietic support for HSCs, finding a potentially effective treatment modality for AA. Herein, we chose umbilical cord-derived MSCs to explore and screen out an optimal serum-free culture system and observed hematopoietic reconstruction in mouse models of AA treated with MSCs. Furthermore, we also preliminarily explored the mechanism of the immunomodulatory and hematopoietic support of MSCs to provide theoretical support in AA mouse models for the clinical application of MSCs in patients with AA.

## Materials and methods

### Mice

BALB/c (H2-2d) mice (sixty in total) were purchased from Dossy Experimental Animals CO., LTD. (Chengdu, China), and DBA/2 (H2-2d) mice (six in total) were purchased from Vital River Laboratory Animal Technology Co., Ltd. (Beijing, China). All mice were female and 6–8 weeks old, and their weight were about 18–23 g. BALB/c mice were numbered and then, randomly assigned to each group using a random number table. As lymphocytes donors, DBA/2 mice were anesthetized with isoflurane and dislocated at the neck and executed. All mice were housed in a laminar flow chamber (specific pathogen free, SPF) at the West China Science and Technology Park. The animal experimental procedure was approved by and compatible with the ethics guidelines of the Institutional Animal Care and Treatment Committee of West China Hospital. The reporting of animal experiments in our study adhered to the Animal Research: Reporting of In Vivo Experiments (ARRIVE) guidelines [19].

### Primary culture of umbilical cord-derived MSC

Wharton's jelly was obtained after removing blood vessels and amniotic membranes in the umbilical cord from fetuses in normal delivery. Tissues from donors followed by the written informed consent. The study met the Code of Ethical Principles for Medical Research Involving Human Subjects of the World Medical Association (Helsinki Declaration). Wharton's jelly tissues were cut into small pieces and cultured in three serum-free media, including the Mesencult-XF purchased from STEMCELL Technologies (Vancouver, Canada), the StemPro MSC SFM CTS purchased from Gibco (CA, USA), and the MCL (A home-made MSC medium based on our previous experiments) [20, 21]. The components of the home-made medium in this study were Dulbecco's Modified Eagle Medium/Nutrient Mixture F-12 (DMEM/F-12), 5% commercialized human platelet lysate, 10 ng/ml recombinant endothelial growth factor (EGF) and 10 ng/ml recombinant basic fibroblast growth factor (bFGF). Animal serum and antibiotics were not added to this medium.

### MSC identification by flow cytometry

The logarithmically grown MSCs were collected and resuspended to 100 μl. Then, 20 μl of the following antibodies was added in the tube: anti-Human IgG1-FITC, IgG1-PE, CD45-FITC, CD90-FITC, CD105-FITC, HLADR-PE (Additional file 1: Table 1), respectively, and incubated for 20 min at room temperature. MSCs were washed and resuspended to 200 ul, and detected with flow cytometry (FC500, Beckman Coulter, Inc., Brea, CA, US).

### Multidirectional differentiation ability of MSCs

P3 MSCs were collected and added to a 6-well plate with a cell count of 1 × 10^5^ per well. After 48 h, the medium was removed, and osteogenesis induction medium, chondrogenesis induction medium, and lipogenesis induction medium (the ingredients in Additional file 1: Tables 2–4) were added to each well, respectively. After 21 days, the media were removed and MSCs were washed by PBS. MSCs were fixed with 4% paraformaldehyde for 30 min and stained with Alizarin Red working solution (Sigma-Aldrich, Inc, St Louis, MO, US) at room temperature for 30 min for osteogenesis induction. MSCs were treated with 1% hydrochloric acid for 5 min and stained with Alcian Blue working solution (Sigma-Aldrich, Inc, St Louis, MO, US) at room temperature for 30 min for chondrogenesis induction. MSCs were fixed with 4% paraformaldehyde for 30 min and stained with Oil Red O working solution (Sigma-Aldrich, Inc, St Louis, MO, US) at room temperature for 30 min for lipogenesis induction.

### MSC functional assay in different media

P3 MSCs were inoculated in a 6-well plate for 72 h, and then, the supernatant was collected and centrifuged for sample preparation (1000 g, 5 min). Enzyme-linked immunosorbent assay (ELISA) kits (R&D Systems Inc., Minneapolis, MN, USA) are used to detect the levels of hepatocyte growth factor (HGF), ANG-1, prostaglandin E2 (PGE2), and TGF-β1. Subsequently, MSCs treated with mitomycin were co-cultured with peripheral blood mononuclear cells (PMNCs) for three days, and then, IFN-γ and TNF-α were detected via ELISA after supernatant was collected and centrifuged (1000 g, 5 min). The ELISA procedure is performed by referring to the instructions of the kit and the literature we previously published [20, 21].

### Establishment of AA mouse model

All mice were randomly divided into three groups: the irradiation, AA, and MSC groups. The mice in the irradiation group were only irradiated; the mice in the AA and MSC groups were based on the mouse model of AA which was established by referring to the method of Zhou and Huang et al. [22, 23]. The lymph nodes were removed from the thymus, neck, submaxilla, axilla, inguinal and mesenteric areas of the DBA/2 mice executed by neck amputation, ground and then, filtered through a sieve to form a single cell suspension. The activity of these cells was identified as 95% or more by using Trypan Blue. Four hours after irradiation at a dose of 5.5 Gy, BALB/c mice in the AA and MSC groups received 1 × 10^6^ mixed cells from the thymus and lymph nodes of DBA/2 mice through the tail vein according to a published study [24]. Blood count and BM biopsy were used to evaluate the success of AA mouse models. The mice in the MSC group receive MSC infusion, while the mice in the irradiation and AA groups do not. All mice were recorded for weight, activity, appearance, diet, and death until the end of the experiment on day 28. On days 7, 14, 21, and 28, the mice in the three groups had transorbital blood collection. These blood samples were used for the hemoglobin (Hb), white blood cell (WBC), and platelet (PLT) count by Automatic Hematology Analyzer (HEMAVET), and Treg and VEGF detection.

### Treg detection by flow cytometry

CD4 (5 µl) and CD25 (5 µl) (Additional file 1: Table 1) were added to fresh MNCs (100 µl) that were from collected peripheral blood by disruption of red blood cells and incubated for 30 min at 4 °C in the dark. Foxp3 was stained by Intracellular Fixation & Permeabilization Buffer Set Kit and Foxp3/Transcription Factor Staining Buffer Set Kit (eBioscience, San Diego, CA, US). Fixation/Permeabilization Concentrate: Fixation/Permeabilization Diluent (v/v: 1/3) was prepared and added to the washed cells for membrane rupture (incubation for 60 min at 4 °C in the dark). After cell suspension was washed (2000 rpm, 5 min) by 1 × permeabilization buffer, Foxp3-PE (4 µl, Additional file 1: Table 1) was added and incubated for 60 min at 4 °C in the dark. Next, the cell suspension was washed by permeabilization buffer (2500 rpm, 7 min) twice and resuspended to 200 µl for detection by flow cytometry (FC500, Beckman Coulter, Inc., Brea, CA, US).

### VEGF detection by ELISA

On days 7, 14, 21 and 28 after irradiation, blood was collected from each group of mice for centrifugation (4 °C, 10000 g, 10 min), and the plasma was collected and frozen at -80 °C for storage. VEGF detection by mouse VEGF ELISA Kit (Multisciences Biotech Co., Ltd., Hangzhou, China) was performed referring to the instruction of the kit and the literature we previously published [20, 21].

### HE staining of mouse BM

The femur was dissected from dead or sacrificed mice on day 28 for hematoxylin and eosin (HE) staining to evaluate the hyperplasia of BM. Femur was fixed in 10% neutral formalin. Paraffin sections were immersed in xylene for 5 min (thrice), placed in 100% anhydrous ethanol for 3 min (twice), then placed in 90–70% ethanol at all levels for 3 min each in turn; rinsed in water for 3 min, placed in double-distilled water for 3 min, and then, rinsed in PBS for 3 times (3 min each) to dewax the hydrated tissue sections. The sections were stained in hematoxylin staining solution for 3–5 min, washed in double distilled water, and then, divided into differentiation solution for 5 s, rinsed in blue with running water for 5–10 min, and washed in double distilled water. Next, sections were placed in 75% ethanol for 5 s, then put into eosin staining solution for 20 s, and washed once with double distilled water. And then sections were placed sequentially in ethanol at 75–95% levels for 1 min each, then in anhydrous ethanol for 1 min and dipped in xylene for 2 min to be transparent (twice). Finally, the sections were dried and sealed with neutral resin and observed under the microscope.

### Statistical analysis

The statistical data are presented as percentages (%), and the measurement data are presented as the mean ± standard deviation (SD). One-way analysis of variance (ANOVA) followed by Tukey's post-test was used to test the significance of the means of each group. Two-sided *P* values < 0.05 were considered significant. The required number of mice per group was calculated based on the significance test level (*α* = 0.05). All analyses were performed with GraphPad Prism 8.0 software (GraphPad Software, San Diego, CA, USA).

## Result

### Primary culture of umbilical cord-derived MSCs

**Fig. 1:**
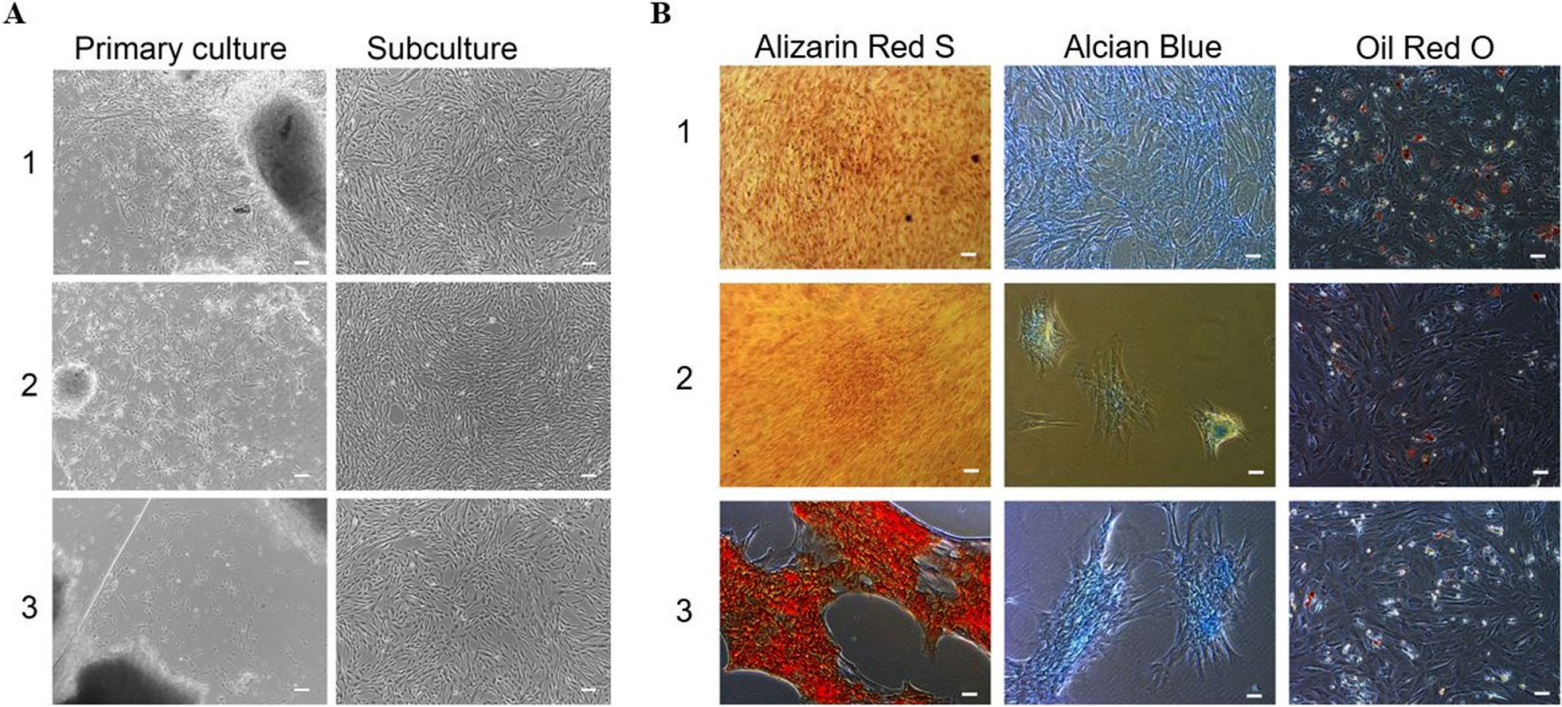
Morphology and differentiation of MSCs. **A** Morphology of MSCs in different media, scale bar: 25 μm; **B** Differentiation of MSCs in different media, scale bar: 50 μm (1. Mesencult-XF, 2. MCL, 3. StemPro MSC SFM CTS)

After five days of culture, long spindle-shaped cells crawl out from the edge of the umbilical cord in these three media. On day 12, the spiral growth of cells could be observed. No difference was found among the three media in terms of attachment, growth time, and morphology of MSCs (Fig. 1A).

### MSC functional assay in different media

MSCs in the three media were examined for osteogenic, chondrogenic, and lipogenic abilities. MSCs in the Mesencult-XF and MCL showed strong chondrogenesis and lipogenesis, and weak osteogenesis while MSCs in the StemPro MSC SFM CTS presented osteogenesis and chondrogenesis, and weak lipogenesis (Fig. 1B).

**Fig. 2:**
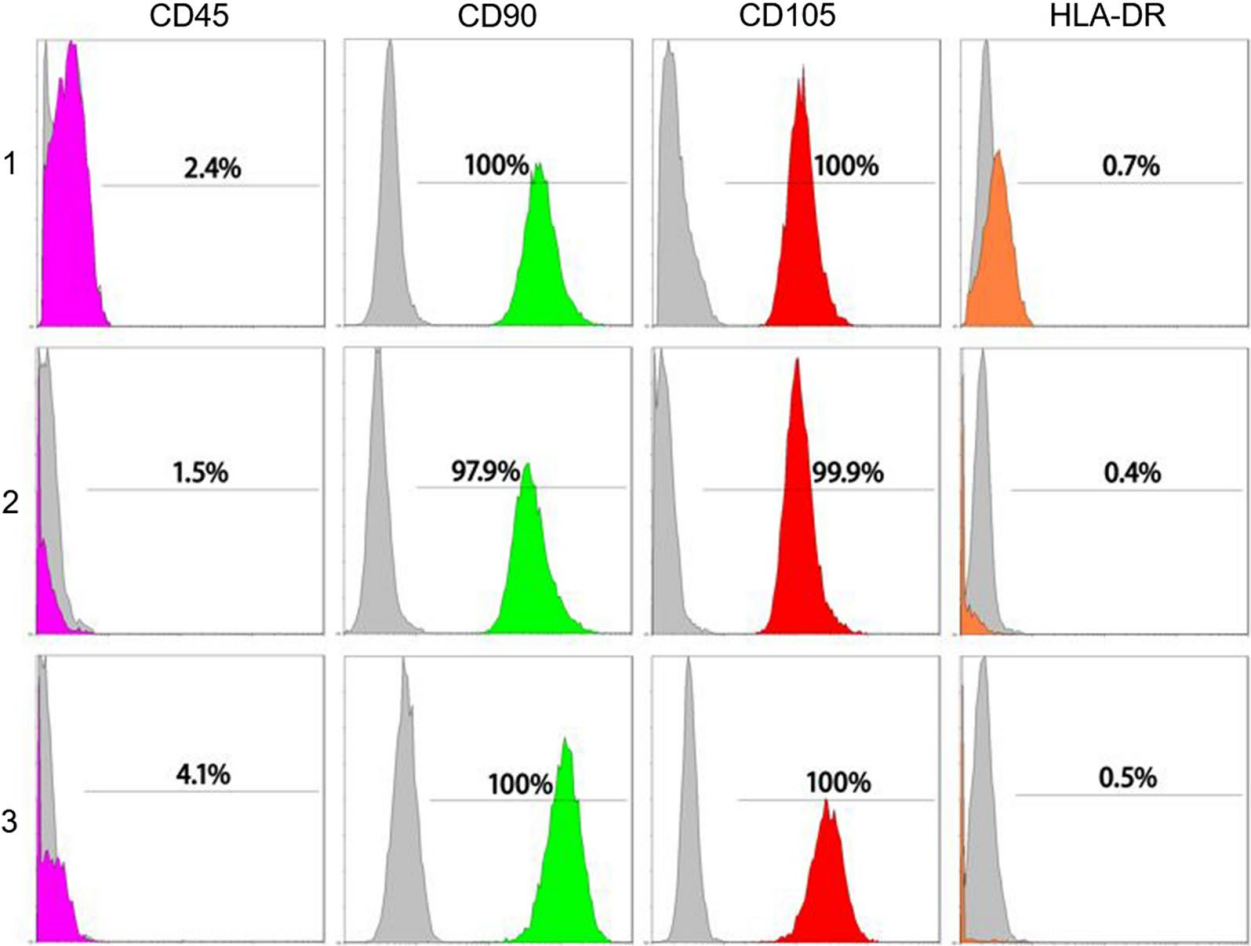
Immunophenotype of MSCs in different media (1. Mesencult-XF; 2. MCL; 3. StemPro MSC SFM CTS)

P3 MSCs cultured in the three media all strongly expressed CD90 and CD105 and did not express CD45 and HLA-DR. There was no difference in antigen expression among the three media (Fig. 2).

**Fig. 3:**
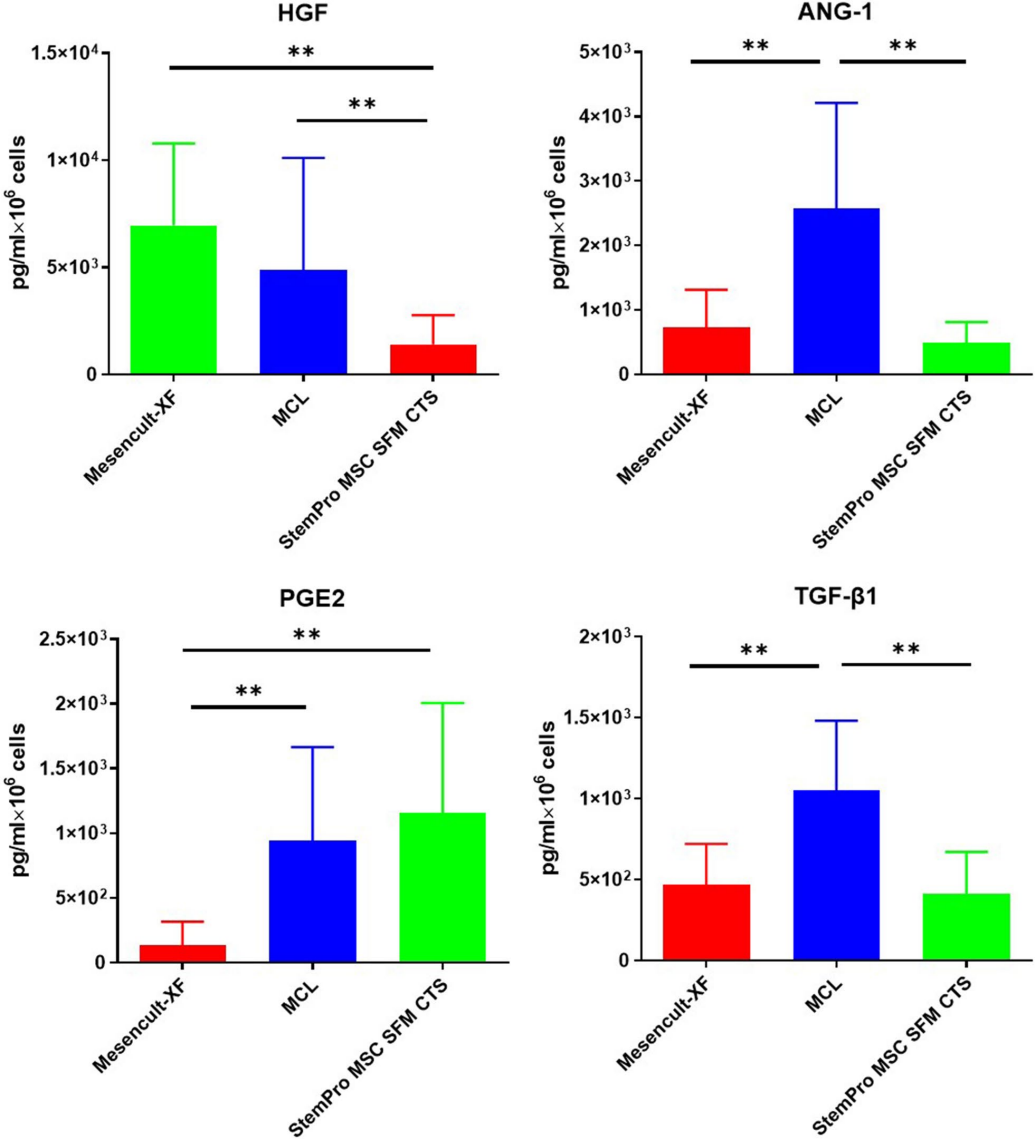
Cytokines (HGF, ANG-1, PGE2, and TGF-β1) secreted by MSCs in different media (**P < 0.01)

The levels of HGF, ANG-1, PGE2, and TGF-β1 secreted by MSCs in the three media were detected by ELISA. As shown in Fig. 3, more HGF was produced in the Mesencult-XF and MCL than in the StemPro MSC SFM CTS (*P* < 0.01); the level of ANG-1 was higher in the MCL than in the Mesencult-XF and StemPro MSC SFM CTS (*P* < 0.01); PGE2 in the MCL and StemPro MSC SFM CTS was higher than that in the Mesencult-XF (*P* < 0.01); and TGF-β1 was secreted more in the MCL than in the Mesencult-XF and StemPro MSC SFM CTS (*P* < 0.01).

IFN-γ and TNF-α levels were measured by ELISA after the MSCs treated with mitomycin C were co-cultured with PMNCs at ratios of 1:10 and 1:5 for 3 days, respectively. The results showed that MSCs in the MCL and StemPro MSC SFM CTS exhibited a stronger inhibition on PMNC-secreted IFN-γ and TNF-α than those in the Mesencult-XF at ratios of 1:10 and 1:5 (*P* < 0.05). There was no significant difference between the MCL and StemPro MSC SFM CTS on the inhibition of MSCs, and the ratio of MSCs: PMNCs did not significantly affect the inhibition of MSCs (Tables 1 and 2). Thus, MCL is a suitable choice for subsequent animal experiments due to its ability to secrete higher levels of immunomodulatory and hematopoietic cytokines and its stronger inhibitory effect on IFN-γ and TNF-α secretion, which have important pathological effects in AA.

**Table 1:** Inhibition of MSCs on IFN-γ released by PMNCs (pg/ml) (X ± SD)

	MSC:PMNC	IFN-γ	MSC:PMNC	IFN-γ
Control		1119.50 ± 234.52		1119.50 ± 234.52
Mesencult-XF	1: 10	1055.55 ± 189.33*	1: 5	653.06 ± 94.85^△^
MCL	1: 10	218.99 ± 37.39*	1: 5	207.35 ± 30.65^△^
StemPro MSC SFM CTS	1: 10	139.59 ± 29.85*	1: 5	148.53 ± 31.28^△^

*P < 0. 05 Mesencult-XF vs MCL, Mesencult-XF vs. StemPro MSC SFM CTS

△*P* < 0. 05 Mesencult-XF vs MCL, Mesencult-XF vs. StemPro MSC SFM CTS

**Table 2:** Inhibition of MSCs on TNF-α released by PMNCs (pg/ml) (X ± SD)

	MSC:PMNC	TNF-α	MSC:PMNC	TNF-α
Control		695.86 ± 110.11		695.86 ± 110.11
Mesencult-XF	1: 10	663.29 ± 121.40*	1: 5	588.91 ± 100.30^△^
MCL	1: 10	485.12 ± 61.11*	1: 5	389.35 ± 46.72^△^
StemPro MSC SFM CTS	1: 10	423.86 ± 50.33*	1: 5	335.89 ± 56.76^△^

*P < 0. 05 Mesencult-XF vs MCL, Mesencult-XF vs. StemPro MSC SFM CTS

△*P* < 0. 05 Mesencult-XF vs MCL, Mesencult-XF vs. StemPro MSC SFM CTS

### The efficacy of MSC for the AA mouse model

**Fig. 4:**
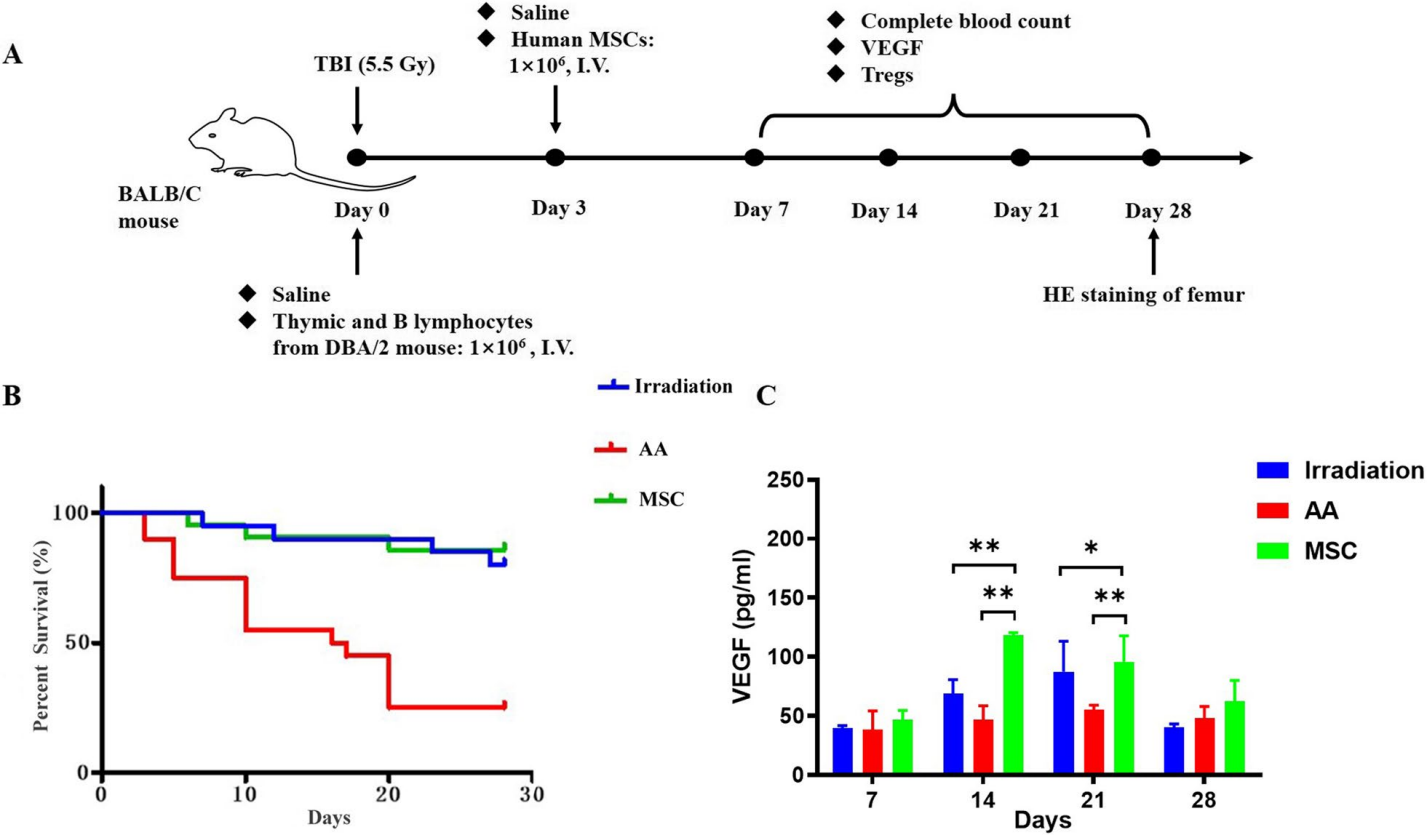
Establishment of AA mouse model and treatment of MSCs in cultured in the MCL medium. **A** Schematic illustration of the animal experiments; **B** Survival curve of mice in the three groups; **C** VEGF levels in the three groups of mice at different time points (*P < 0.05; **P < 0.01)

As described in Materials and methods, we have successfully established the AA mouse model. All mice were included in our study and randomly divided into three groups of 20 mice each: the irradiation, AA, and MSC groups. Three days later, the mice in the irradiation and AA groups were treated with saline and those mice in the MSC group were treated with umbilical cord-derived MSCs (Fig. 4A). In the irradiation group, the mice showed a slight decrease in body weight 1 week after irradiation and returned to normal at 3 weeks, and the 28-day overall survival rate was 80%. In the AA group, the mice lost weight 5 days after irradiation and did not regain weight significantly during the experiment, and the 28-day overall survival rate was 35%. In the MSC group, weight changes were similar to those in the irradiation group, and the 28-day overall survival rate was 85% (Fig. 4B).

The levels of VEGF in the three groups on days 7, 14, 21, and 28 are displayed in Fig. 4C. In the irradiation and MSC groups, the level of VEGF increased on days 14 and 21 and decreased on day 28. In the AA group, no significant change in VEGF was found from day 7 to day 28. Between the irradiation and AA groups, no difference in VEGF was found. Between the irradiation and MSC groups, there were significant differences on days 14 and 21 (*P* < 0.01). Between the AA and MSC groups, differences also existed on days 14 (*P* < 0.01) and 21 (*P* < 0.01).

**Fig. 5:**
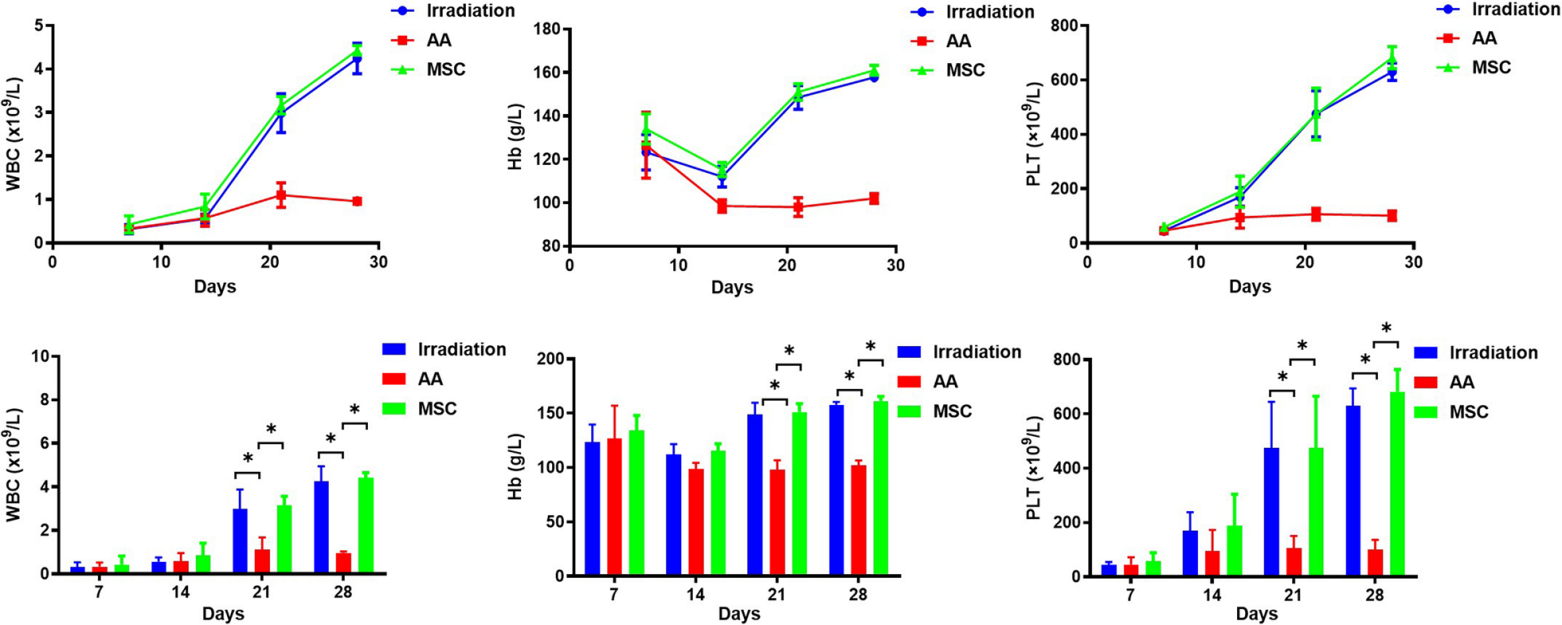
WBC, Hb and PLT counts of mice in the three groups at different time points **A** WBC count; **B** Hb count; **C** PLT count (*P < 0.05)

As illustrated in Fig. 5, WBC, Hb, and PLT counts all decreased at the early stage. Subsequently, WBC, Hb, and PLT counts increased quickly in the irradiation and MSC groups from day 14 while those in the AA group were still at low levels until day 28. Compared with the irradiation group and MSC group, WBC, Hb, and PLT counts were significantly lower in the AA group on days 21 and 28.

**Fig. 6:**
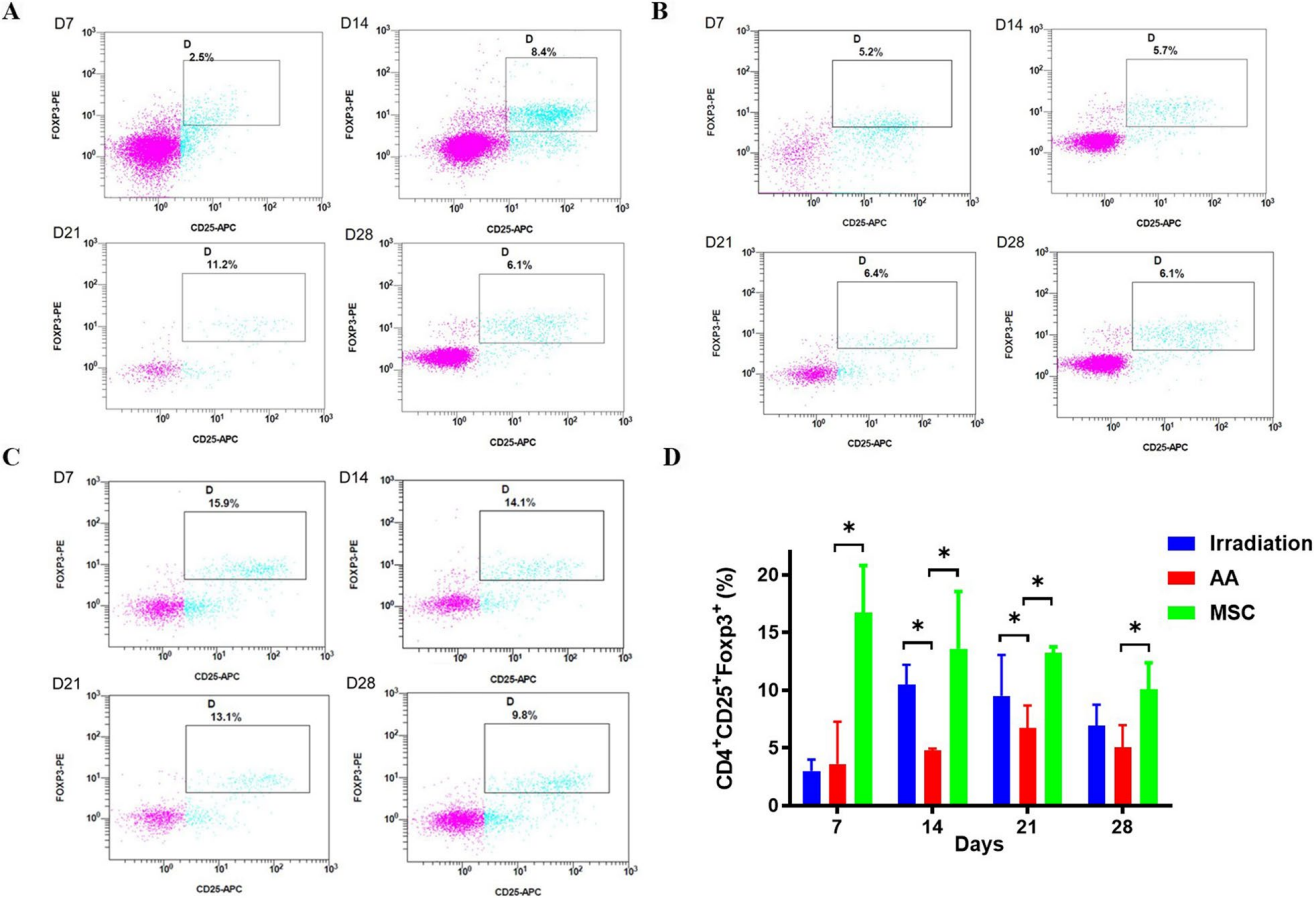
Tregs of mice in the irradiation, AA, MSC groups at different time points. **A** Flow cytometry analysis of Treg change in the irradiation group; **B** Flow cytometry analysis of Treg change in the AA group; **C** Flow cytometry analysis of Treg change in the MSC group; **D** Quantitative comparison of Treg differences among the three groups (*P < 0.05)

Next, we detected the proportion of Tregs on days 7, 14, 21, and 28 in the three groups via flow cytometry. As shown in Fig. 6, the Treg ratio of the AA group was significantly lower than that of the irradiation group on days 14 and 21 (*P* < 0.05) and was also significantly lower than that of the MSC group on days 7, 14, 21, and 28 (*P* < 0.05). And the Treg ratio of the MSC group on day 7 was higher than that of the irradiation group (*P* < 0.05).

**Fig. 7:**
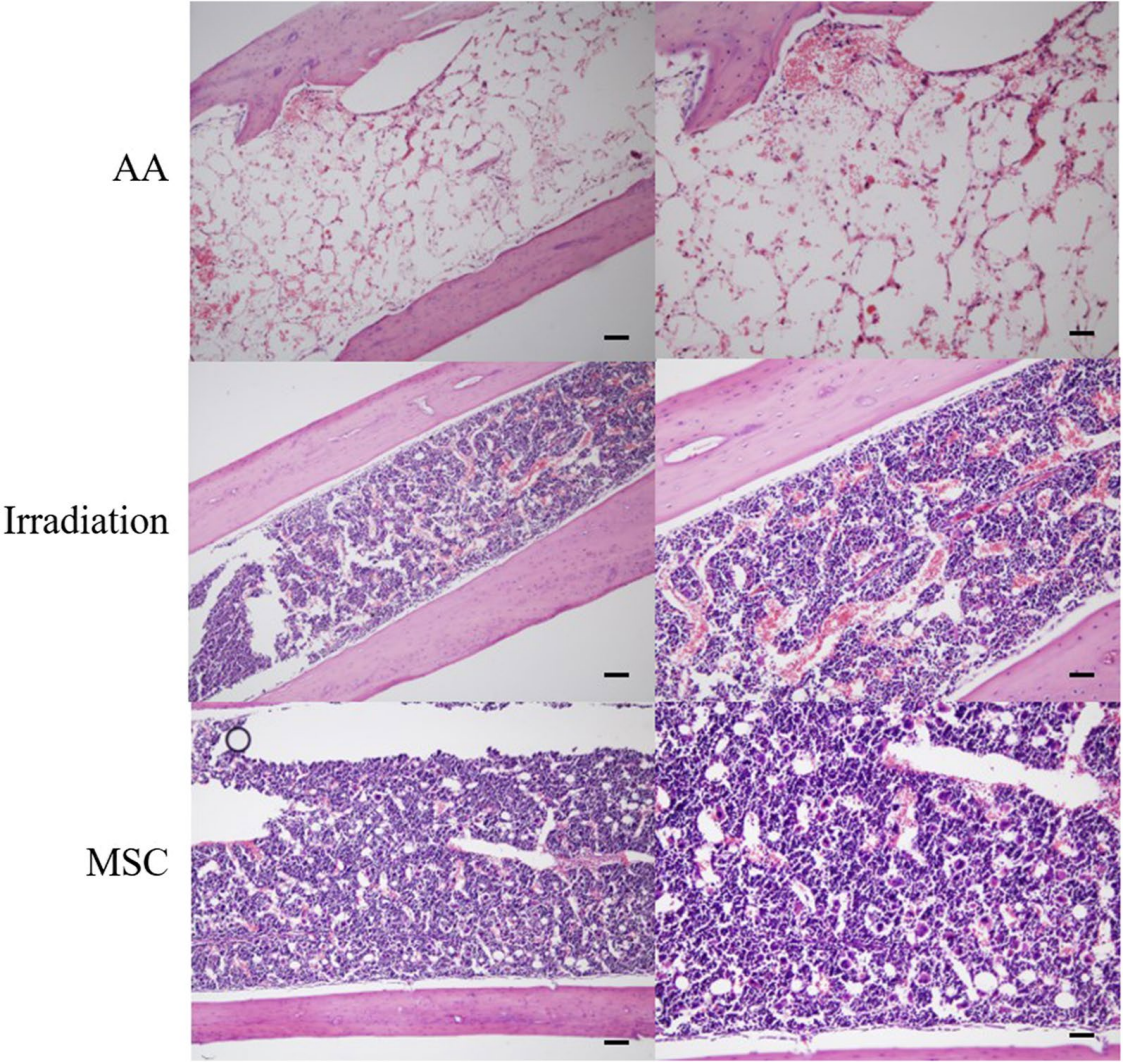
HE staining of the femur of mice in the irradiation, AA, MSC groups at different magnifications (Scale bar at left: 50 μm; Scale bar at right: 100 μm)

The femurs of mice were stained with HE to investigate the effect of MSCs on BM hematopoiesis. As shown in Fig. 7, BM was significantly dysplastic, hematopoietic tissues were reduced, and many fat vacuoles and exudates were observed in the AA group of mice. In the irradiation and MSC groups, BM proliferation was hyperplastic, with many karyocytes and little adipose tissue. Compared with those of the irradiation group, BM in the MSC group has denser karyocytes.

## Discussion

In 2006, the Mesenchymal and Tissue Stem Cell Committee of the International Society for Cellular Therapy (ISCT) defined three minimum criteria for the identification of MSCs as follows: (1) adherent growth of MSCs under standard culture conditions; (2) expression of CD105, CD73, and CD90, and non-expression of CD45, CD34, CD14, CD11b, CD79a, CD19 and HLA-DR on the MSC surface; and (3) osteogenic, lipogenic and chondrogenic differentiation potential of MSCs in vitro [25].

In our study, serum-free media were chosen to culture MSCs due to the need for clinical safety in the future [26, 27]. A large number of MSCs were harvested via umbilical cord tissue block adherence in kinds of media. By examining the differentiation capacity of P3 MSCs and the immunophenotype of the cell surface, cells from all three media showed the properties of MSCs.

Tregs are a subset of CD4 + T cells that play an important role in the negative regulation of the immune response and autoimmune tolerance [28], which is important in the pathogenesis of AA. Some studies have reported that excessive IFN-γ and TNF-α inhibit Tregs and damage HSCs in BM in AA patients [29, 30]. It has also reported decreased various cytokines supporting hematopoiesis, such as SCF, VEGF, TGF-β, and ANG-1, which reduced osteoblast capacity and further aggravated hematopoietic damage [18, 31, 32]. Furthermore, VEGF can promote the growth of vascular endothelial cells and increase the permeability of microvessels, thus promoting vascular neovascularization [33]. In addition, HGF, ANG-1, PGE2, and TGF-β1, which play important roles in maintaining the hematopoietic microenvironment and promoting cell growth, proliferation, and differentiation as well as immune regulation, were used as representative cytokines in MSC culture. Therefore, we expected a medium that can reduce the levels of IFN-γ and TNF-α and promote the secretion of hematopoietic cytokines.

Before our study, numerous studies have investigated the efficacy of human MSCs in the treatment of AA mouse model. There were many sources of MSCs in these studies, including BM, umbilical cord, adipose tissue, gingiva tissue, and multi-placenta pooled cells, all of which invariably have confirmed that MSCs can help restore hematopoietic reconstitution and improve the prognosis [34–39]. Nevertheless, these previous studies did not go further to optimize the culture system of MSC for its excellent hematopoietic function of repairing bone marrow, which was also the most significantly different from our study. Our study is the first to screen the culture system for highly active MSCs. It was also demonstrated that umbilical cord-derived MSCs cultured in the MCL medium could be expanded to be capable of secreting higher level hematopoietic support cytokines and more potent inhibition of IFN-γ and TNF-α secretion compared with other candidate culture systems.

In the animal experiment, we found that Tregs increased rapidly in a short period in the mice of the MSC group and were even more abundant than those in the irradiation group. Some studies have reported that MSCs regulate the differentiation of CD4 + T cells into Tregs by inhibiting the differentiation of CD4 + T cells into Th17 cells and inducing Th17 cells to express Foxp3, and directly promote the proliferation and differentiation of Tregs by regulating the expression of cytokines such as TGF-β, IL-6 and IL-10 [40–42]. Thus, we supposed that the proliferation and differentiation of Tregs in the MSCs group of mice were both promoted, probably for the purpose of greater suppression of immune disorders in mice after AA modeling. In terms of the VEGF level, it was found to be significantly higher in the MSC group than that in the other two groups, which indicated that these MSCs also had an excellent ability to increase VEGF level and thus, improve the hematopoietic microenvironment in BM.

Although our study is innovative in the MSC culture system, some limitations should be also noted. First, the MSC cultured in the other two media should be also subjected to animal experiments to clarify their efficacy in vivo. Second, Treg study was just limited to a phenotyping by flow cytometry, but no Treg functional tests or Treg induction tests. We plan to remedy these deficiencies in future experiments.

## Conclusion

In our study, umbilical cord-derived MSCs cultured in the three media conformed to the general characteristics of MSCs. The MCL medium was superior in the secretion of cytokines with hematopoietic and immunomodulatory properties and a stronger ability to inhibit IFN-γ and TNF-α. In the mouse experiment, the results of blood counts and BM biopsies suggested that MSCs could restore hematopoiesis, and the Treg ratios and VEGF levels in the MSC group were higher than those in the other two groups. In conclusion, umbilical cord-derived MSCs showed an excellent efficacy in the treatment of AA and have the potential to reconstruct hematopoiesis clinically in the future.

### Supplementary Information

**Additional file 1: Table 1.** List of antibodies used in Flow cytometry.** Table 2.** Formulation of osteogenesis induction solution.** Table 3.** Formulation of chondrogenesis induction solution.** Table 4.** Formulation of lipogenesis induction solution.

## Abbreviations

AA: Aplastic anemia
IFN-γ: Interferon-γ
TNF-α: Tumor necrosis factor-α
HSCs: Hematopoietic stem cells
MSC: Mesenchymal stem cell
PMNC: Peripheral blood mononuclear cell
ELISA: Enzyme-linked immunosorbent assay
HGF: Hepatocyte growth factor
ANG-1: Angiogenesis factor-1
PGE2: Prostaglandin E2
TGF-β1: Transforming growth factor-beta1
VEGF: Vascular endothelial cell growth factor
Tregs: Regulatory T cells
SCF: Stem cell factor
IST: Immunosuppressive therapy
BM: Bone marrow
Hb: Hemoglobin
WBC: White blood cell
PLT: Platelet

## **Declarations**

### Availability of data and materials

Please contact the author for data and materials.
