# Supplementary material for: Umbilical cord-derived mesenchymal stem cells cultured in the MCL medium for aplastic anemia therapy
Source: Stem Cell Res Ther. 2023 Aug 30;14:224. doi: 10.1186/s13287-023-03417-1 (PMC10470151; doi:10.1186/s13287-023-03417-1)
Supplement: Supplementary file 1 — Additional file 1: Table 1. List of antibodies used in Flow cytometry. Table 2. Formulation of osteogenesis induction solution. Table 3. Formulation of chondrogenesis induction solution. Table 4. Formulation of lipogenesis induction solution. [file 13287_2023_3417_MOESM1_ESM.docx]

| **Target** | **Type** | **Fluorescence** | **Company** |
| --- | --- | --- | --- |
| IgG1 | Anti-Human | FITC | BD Biosciences |
| IgG1 | Anti-Human | PE | BD Biosciences |
| CD45 | Anti-Human | FITC | BD Biosciences |
| CD90 | Anti-Human | FITC | BD Biosciences |
| CD105 | Anti-Human | PE | BD Biosciences |
| HLA-DR | Anti-Human | PE | BD Biosciences |
| CD4 | Anti-Mouse | FITC | eBioscience |
| CD25 | Anti-Mouse | APC | eBioscience |
| Foxp3 | Anti-Mouse/Rat | PE | BD Biosciences |

**Table 1. List of antibodies used in Flow cytometry.**

**Table 2. Formulation of osteogenesis induction solution.**

| **Ingredients** | **Concentration** |
| --- | --- |
| FBS (fetal bovine serum) | 10% |
| Dexamethasone | 10-8 M |
| L-Ascorbic acid | 50 μM |
| β-Glycerophosphate | 10 mM |
| 1, 25-Dihydroxyvitamin D3 | 0.01 μM |
| penicillin | 100 units/ml |
| streptomycin | 100 μg/ml |

**Table 3. Formulation of chondrogenesis induction solution.**

| **Ingredients** | **Concentration** |
| --- | --- |
| BSA (bovine serum albumin) | 10^-7^ M |
| Dexamethasone | 10^-8^ M |
| L-Ascorbic acid | 50 μM |
| sodium pyruvate | 100 mg/ml |
| TGF-β  Fibroblast growth factor β  ITS-Plus Media Supplement  proline | 10 ng/ml  1 ng/ml  6.25 mg/ml  40 mg/ml |
| penicillin | 100 units/ml |
| streptomycin | 100 μg/ml |

**Table 4. Formulation of lipogenesis induction solution.**

| **Ingredients** | **Concentration** |
| --- | --- |
| FBS (fetal bovine serum) | 10% |
| Dexamethasone | 1 μM |
| Insulin | 5 μg/ml |
| IBMX (3-isobutyl-1-methylxanthine) | 0.5 mM |
| Indomethacin | 0.2 mM |
| penicillin | 100 units/ml |
| streptomycin | 100 μg/ml |
